# Prognostic factors and outcome of adult allogeneic hematopoietic stem cell transplantation patients admitted to intensive care unit during transplant hospitalization

**DOI:** 10.1038/s41598-019-56322-0

**Published:** 2019-12-27

**Authors:** Christian S. Michel, Daniel Teschner, Irene Schmidtmann, Matthias Theobald, Beate Hauptrock, Eva M. Wagner-Drouet, Markus P. Radsak

**Affiliations:** 1 Department of Hematology, Medical Oncology, & Pneumology, University Medical Center of the Johannes Gutenberg University, Mainz, Germany; 2 Institute of Medical Biostatistics, Epidemiology and Informatics (IMBEI), University Medical Center of the Johannes Gutenberg University, Mainz, Germany

**Keywords:** Prognosis, Outcomes research, Respiratory signs and symptoms

## Abstract

Patients undergoing allogeneic hematopoietic stem cell transplantation have a high morbidity and mortality, especially after admission to intensive care unit (ICU) during peri-transplant period. The objective of this study was to identify new clinical and biological parameters and validate prognostic scores associated with ICU, short-and long-term survival. Significant differences between ICU survivors and ICU non-survivors for the clinical parameters invasive mechanical ventilation, urine output, heart rate, mean arterial pressure, and amount of vasopressors have been measured. Among prognostic scores (SOFA, SAPSII, PICAT, APACHE II, APACHE IV) assessing severity of disease and predicting outcome of critically ill patients on ICU, the APACHE II score has shown most significant difference (*p* = 0.002) and the highest discriminative power (area under the ROC curve (AUC) 0.74). An elevated level of lactate at day of admission was associated with poor survival on ICU and the most significant independent parameter (*p* < 0.001). In our cohort kidney damage with low urine output has a highly relevant impact on ICU, short- and long-term overall survival. The APACHE II score was superior predicting ICU mortality compared to all other tested prognostic scores for patients on ICU during peri-transplant period.

## Introduction

Allogeneic hematopoietic stem cell transplantation (allo-HSCT) offers a potential curative treatment for a variety of hematological diseases. However, the transplantation procedure is commonly connected to a variety of transplant-related factors such as patient age, intensity of conditioning, type of graft, presence of infections, many of which have significantly changed over the past decades^1^. The most common reasons for intensive care unit (ICU) admission after allo-HSCT are respiratory failure and septic shock. Other reported reasons for ICU admission include cardiac dysfunction, neurological disorders, and severe hemorrhage^2,3^. Despite advances in intensive care treatment and allo-HSCT, outcome of patients who need to be treated on ICU after allo-HSCT remains poor. After admission to ICU, in-hospital mortality of allo-HCST patients ranges from 53 to 75% with a 1-year overall survival of 15 to 28%^4^, much lower than the general overall 1-year survival of 60–70% in allo-HSCT recipients^4,5^. Unfortunately, several previously published studies often combined allogeneic and autologous stem cell transplanted patients and the related prognostic ICU scores like Sequential Organ Failure Assessment (SOFA)^6^, Simplified Acute Physiology Score II (SAPS II)^7^, and the Acute Physiology And Chronic Health Evaluation score II and IV (APACHE II and IV)^8–10^. Some studies found that these scoring systems underestimate the mortality of allo-HSCT patients, and yet others found that they are useful for estimating mortality in the ICU setting^9,11–13^. Recently, a new prognostic index tailored for critically ill allo-HSCT patients has been developed, the Prognostic Index for Critically Ill Allogeneic Transplantation Patients (PICAT score)^14^.

The primary objective of the study was to assess short-and long-term outcomes, to identify new clinical and biological predictors as well as the comparison of new and established prognostic factors associated with ICU, hundred day and 1-year mortality for allo-HSCT patients admitted to ICU during the peri-transplant period.

## Results

### Patient characteristics

During the study period 544 patients received a first allo-HSCT of whom 81 (14.9%) patients had to be transferred to ICU during their transplant hospitalization. Three patients who were admitted on ICU during conditioning chemotherapy and died before allo-HSCT were excluded from further analysis. The main characteristics of the total cohort and the subgroups (ICU survivors vs. ICU non-survivors) are shown in Table 1. Median age at time of hematopoietic stem cell transplantation was 54.4 years (interquartile range (IQR) 45.2–61.1). Main underlying diseases were acute leukemias with 75.6% as well as myeloproliferative diseases and myelodysplastic syndrome (MDS) with 16.7%. The hematopoietic malignancy had no influence on ICU survival (ns).

**Table 1:** Baseline characteristics of all patients admitted to ICU.

	All patients, n = 78		ICU survivors, n = 44		ICU non-survivors, n = 34		p-Value
Sex, male, n (%)	45	(57.7)	23	(52.3)	22	(64.7)	0.270
Age, years, median (IQR)	54.4	(45.2–61.1)	51.4	(45.4–60.8)	58.5	(46.6–60.8)	0.579
BMI, median (IQR)	23.7	(21.6–26.9)	23.1	(21.5–26.2)	24.5	(22.3–27.4)	0.247
Underlying disease -Acute leukemia, n (%) -Myeloproliferative disease and MDS, n (%) -Lymphoma and Multiple Myeloma, n (%) -Aplastic anemia, n (%)							0.125
	59	(75.6)	31	(70.5)	28	(82.4)	
	13	(16.7)	10	(22.7)	3	(8.8)	
	4	(5.1)	1	(2.3)	3	(8.8)	
	2	(2.6)	2	(4.5)	0	(0)	
Remission status -Refractory disease, n (%) Conditioning regimen -MA, n (%) -RIC and NMA, n (%) -FLAMSA-RIC, n (%)	23	(29.5)	14	(31.8)	9	(26.5)	0.608 0.965
	24 33 21	(30.8) (42.3) (26.9)	13 19 12	(29.5) (43.2) (27.3)	11 14 9	(32.4) (41.2) (26.4)	
Source of stem cells -Peripheral blood, n (%) -Bone marrow, n (%)	76	(97.4)	42	(95.5)	34	(100)	0.208
	2	(2.6)	2	(4.5)	0	(0)	
Graft -HLA identical sibling, n (%) -HLA matched unrelated donor, n (%) -HLA-mismatched, n (%)	11	(14.1)	7	(15.9)	4	(11.8)	0.339
	53	(68.0)	27	(61.4)	26	(76.4)	
	14	(17.9)	10	(22.7)	4	(11.8)	
CMV -Positive, n (%)	51	(65.4)	29	(70.5)	22	(64.7)	0.912
HCT-CI score, median (IQR)	3	(2.0–5.0)	3	(2.0–5.0)	3	(1.0–5.0)	0.485
Time from Hospital to ICU admission (d), median (IQR) Reason for ICU admission -Sepsis, n (%) -Respiratory failure, n (%) -Cardiac arrest/arrhytmia,n (%) -Post-surgery, n (%) -Neurological disorders, n (%) -Severe bleeding, n (%)	19 28 59 10 3 20 5	(11.8–27.0) (35.9) (75.6) (7.8) (3.8) (25.6) (6.4)	19.5 12 32 6 3 12 5	(11.8–27.0) (27.3) (72.7) (13.6) (6.8) (27.3) (11.4)	18 16 27 4 0 8 0	(13.3–26.8) (47.1) (79.4) (11.8) (0.0) (23.5) (0.0)	0.964 0.071 0.495 0.806 0.120 0.707 0.042

Abbreviations: BMI = Body mass index; MDS = Myelodysplastic syndrome; MA = Myeloablative; RIC = Reduced intensity conditioning; NMA = Non-myeloablative; FLAMSA = Induction chemotherapy before conditioning chemotherapy; HLA = Human Leukocyte Antigen; CMV = Cytomegalivirus; HCT-CI = Hematopoietic Cell Transplantation-specific Comorbidity Index.

About 29.5% of patients were transplanted with refractory disease. Conditioning regimen was myeloablative in 30.8% and 26.9% of patients received FLAMSA as an induction part of the reduced intensity conditioning regimen (FLAMSA-RIC)^15^. The FLAMSA protocol combines a four-day salvage chemotherapy consisting of daily fludarabine, amsacrine and cytarabine, followed by three days of pause with the reduced-intensity conditioning regimen. Peripheral blood stem cells were in 97.4% the source of the stem cell graft and the majority of patients (68%) had a HLA matched unrelated donor. Gender, age, body mass index (BMI), remission status, conditioning regimen, source of stem cells, donor type and cytomegalovirus (CMV) status were not significantly different between ICU survivors and ICU non-survivors.

The median time from hospital admission to ICU admission was 19 days (IQR 11.8–27) and patients had a median HCT-CI score of 3 (IQR 2–5) before allo-HSCT. The single or multifactorial reasons of ICU transfer were respiratory failure (72.7% in ICU survivors, 79.4% in ICU non-survivors, *p* = 0.495), followed by sepsis (27.3% in ICU survivors, 47.1% in ICU non-survivors, *p* = 0.071) and severe neurological disorders (27.3 in ICU survivors, 23.5% in ICU non-survivors, *p* = 0.707). Occurrence of severe bleeding (11.4% in ICU survivors, 0% in ICU non-survivors, *p* = 0.042) was more frequently in ICU survivors. No patient had a recurrent or progressive disease after HSCT when admitted to ICU.

### Clinical and laboratory parameters

During first 24 hours of ICU admission, non-invasive ventilation (NIV) as a first approach was used in 17% of patients and switched in almost half of them to invasive mechanical ventilation (IMV) after NIV failure. Altogether, invasive mechanical ventilation was initiated in 60% of all patients (*n* = 47) with a median Horowitz Index (ratio of partial pressure of oxygen in blood and fraction of oxygen in the inhaled air- PaO_2_/FiO_2_) of 160 (IQR 114.5–273.0). Median duration of IMV was 17 days (IQR 8.5–29) and ICU survivors had lower frequency of IMV (45.5% vs 79.4%, *p* = 0.002).

A total of 68% (*n* = 53) of the patients presented with acute kidney injury (AKI) and RRT was initiated in almost half of these patients (*n* = 24). In addition, level of creatinine was significantly higher in ICU non-survivors (median 2.21 mg/dl vs 1.59 mg/dl, *p* = 0.029) and the urine output in the first 24 hours after admission was significantly lowered (median 1050 ml/d vs 2085 ml/d, *p* = 0.003). Median duration of RRT was 7.5 days (IQR 4–15). Urine output in 24 hours after admission to ICU of 365d survivors was 4055 ml/d (IQR 2048–5015) and of 365d non-survivors 1290 ml/d (IQR 588–2350). The urine output was the most significant clinical and laboratory parameter (*p* < 0.001) in 365d mortality analysis with an AUC of 0.79 (0.66–0.92, 95% CI). Median fluid overload was 4.3 kg (IQR 1.4–6.9) after ICU admission compared to hospital admission but had no impact on ICU survival (ns).

ICU non-survivors suffered from higher heart rate (median 135bpm vs 123bpm, *p* = 0.019) and lower mean arterial blood pressure (median 60.0mmHG vs 63.0 mmHg, *p* = 0.024) than ICU survivors. Treatment with norepinephrine was necessary in 74% of patients and ICU survivors needed lower doses of norepinephrine (median 0.4 mg/h vs 0.9 mg/h, *p* = 0.011). In the same line, ICU non-survivors showed higher level of lactate (median 4.3 mmol/l vs 1.7 mmol/l, *p* < 0.001), while pH (median 7.25 vs 7.31; *p* = 0.015), albumin (median 22 g/l vs 24 g/l, p = 0.026) and bicarbonate (median 18.6 mmol/l vs 20.96 mmol/l, *p* = 0.043) were significantly lowered. Lactate showed best discriminative power among all clinical and laboratory parameters (AUC 0.75, 0.64–0.86 95% CI) for ICU mortality. A cut off value of 3.35 mmol/L has been calculated by using Youden’s *J* statistic. All significant clinical and laboratory parameters are listed in Table 2.

**Table 2:** Prognostic scores, clinical and laboratory parameters.

	All patients, n = 78		ICU survivors, n = 44		ICU non survivors, n = 34		p-Value
**Prognostic Scores**							
-SOFA, median (IQR) -APACHE II, median (IQR) -APACHE IV, median (IQR) -SAPS II, median (IQR) -PICAT, median (IQR)	14 29 102 65 4.0	(12–17) (25–33) (77–126) (53–81) (3.0–5.0)	13.5 27 93 56 4.0	(11.0–16.0) (20–31) (69.0–115.0) (46.8–73.3) (3.0–4.2)	16.0 31 111 70 3.9	(14.0–18.0) (27–37) (93–142) (53–78) (3.1–5.3)	0.010 0.002 0.007 0.131 0.213
**Clinical parameters**							
-Mechanical ventilation, n (%) -RRT, n (%) -Urine output (ml/d), median (IQR) -Heart rate (bpm), median (IQR) -Mean arterial pressure (mmHg), median (IQR) -Norepinephrine (mg/h), median (IQR)	47 24 1530 126 61.0 0.6	(60.3) (30.8) (823–3433) (112–138) (56.0–68.3) (0.0–1.2)	20 10 2085 123 63.0 0.4	(45.5) (22.7) (1008–3983) (105–132) (56.0–72.8) (0.0–0.75)	27 14 1050 135 60.0 0.9	(79.4) (41.2) (288–1775) (119–144) (53.8–63.5) (0.4–2.15)	0.002 0.080 0.003 0.019 0.024 0.011
**Laboratory parameters**							
-Albumin (g/l), median (IQR) -Lactate (mmol/l), median (IQR) -pH, median (IQR) -Bicarbonate (mmol/l), median (IQR) -Creatinine (mg/dl), median (IQR)	23.0 2.3 7.28 19.5 1.97	(20.5–26.5) (1.4–4.6) (7.22–7.37) (17.7–22.2) (1.18–2.75)	24 1.7 7.31 20.9 1.59	(22.0–28.0) (1.1–2.6) (7.25–7.40) (18.7–22.5) (1.10–2.47)	22 4.3 7.25 18.6 2.21	(19.0–25.5) (2.6–6.2) (7.17–7.30) (15.6–21.1) (1.65–2.83)	0.026 <0.001 0.015 0.043 0.029

Abbreviations: RRT = Renal replacement therapy.

Procalcitonin (PCT) at admission was not significantly different between ICU survivors and non-survivors, but a simple linear regression analysis has demonstrated that patients admitted to ICU before and close around the transplant date had higher PCT levels than patients admitted to ICU in later time of transplantation (adjusted R² = 0.242, p < 0.001).

### Prognostic scores

**Figure 1:**
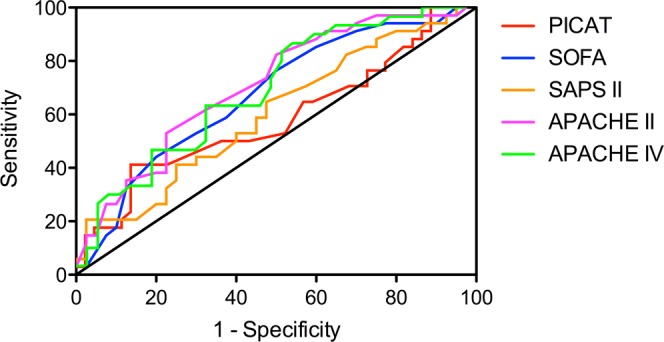
ROC curves for prognostic scores. ROC curves for SOFA, SAPS II, APACHE II, APACHE IV and PICAT score predicting ICU mortality.

Median SOFA and SAPS II score for all patients were 14.0 (IQR 12.0–17.0) and 64.5 (IQR 53.0–81.3), respectively. Our analysis showed significant differences between ICU-survivors and ICU non-survivors for SOFA score, but not SAPS II score (SOFA, median 13.5 vs 16.0, *p* = 0.010; SAPS II, median 56 vs 70, *p* = 0.131). APACHE II and APACHE IV score showed even higher significant differences between ICU-survivors and non-survivors (APACHE II, median 27 vs 31, *p* = 0.002; APACHE IV, median 93 vs 111, *p* = 0.007). Analysis of ROC curves showed that among all prognostic scores APACHE II had the best discriminative power (AUC 0.74, 0.63–0.84 95% CI) followed by SOFA score (AUC 0.70, 0.59–0.82 95% CI). An AUC of 0.74 indicates good discriminative power for APACHE II while the AUC of 0.66 indicates a moderate discriminative power for APACHE IV. In contrast, PICAT score was not significantly different between ICU-survivors and non-survivors (median 4.0 vs 3.9, *p* = 0.102) and had the lowest discriminative power (AUC 0.58, 0.45–0.71 95% CI). In the cohort, ICU mortality of patients with PICAT score 0–2 (n = 9), >2–4 (n = 34) and >4 (n = 35) were 33%, 44% and 46%. AUC for ICU mortality for APACHE II was not significantly larger (*p* = 0.082) than the AUC of the PICAT score (Fig. 1).

The logistic regression model revealed significant effects on 1-year survival for the prognostic scores APACHE II (p = 0.017) and APACHE IV (p = 0.016), whereas SOFA, SAPS II and PICAT score showed no significant effect. APACHE II score showed best discriminative power among all prognostic scores (AUC 0.71, 0.57–0.84 95% CI) for the 1-year survival.

### Short- and long-term survival

**Figure 2:**
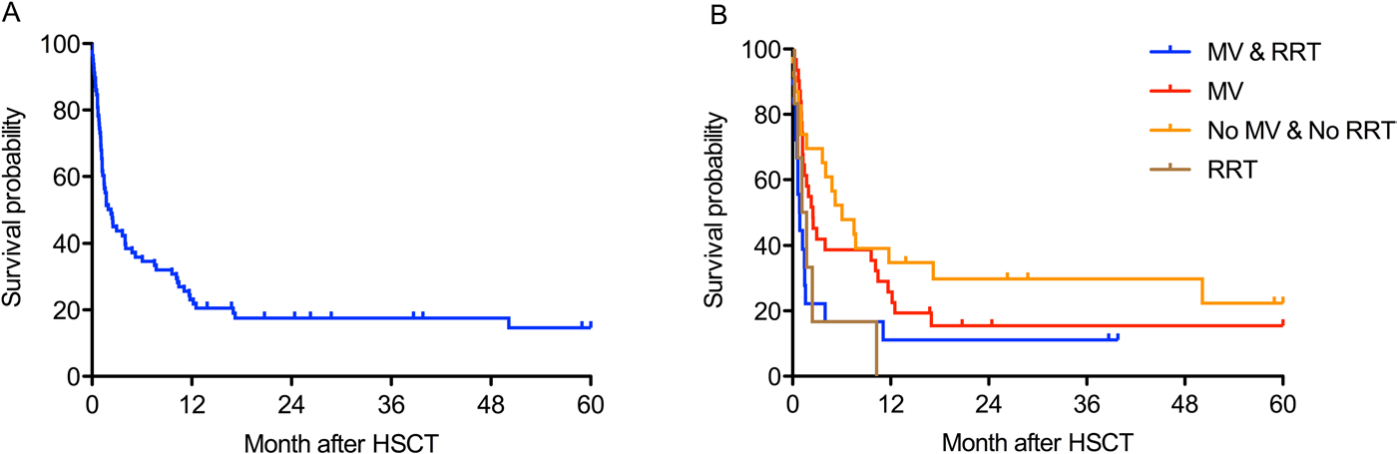
Overall survival (**A**) Overall survival; (**B**) Overall survival of subgroups (MV & RRT, MV, No MV & No RRT, RRT).

Of 78 patients admitted to ICU, 44 patients (56.4%) could be discharged from ICU back to our transplant unit. In the further course, 19 patients had a second admission to ICU in median 17 days after last discharge (IQR 6–73). The 100d and 365d survival rates of all ICU treated patients were 42.3% and 23.1%, respectively. Median overall surivival for all patients was 65 days (IQR 28–351) Fig. 2A. All patients needing RRT (n = 6) with hemodynamic support at the day of ICU admission died within in the first year after transplantation. Patients with IMV and RRT had the second worst survival. Multiple comparison of survival curves have shown that patients requiring no IMV and RRT at the day of ICU admission had a significant better overall survival compared to patients with IMV and RRT (p = 0.013) Fig. 2B. From 44 patients after first ICU admission 13 patients died from severe infectious complications/graft-versus-host disease (GvHD), 12 from relapse of the hematological disease and 4 from severe brain damage. Vast majority (n = 26) was dying in the first year after transplantation, only 3 patients died after the first year.

## Discussion

This study presents the outcome in relation to prognostic scores, as well as clinical and laboratory parameters in a single-center cohort of allo-HSCT patients admitted to ICU during peri-transplant period. The study excluded allo-HSCT patients admitted to ICU in later course after transplantation and inhibited major influence of community-acquired infections, relapse and acute or chronic GvHD complications. Additionally, autologous HSCT patients on ICU were excluded as their outcomes seem to be better and their transplantation- and disease-related factors are different than those of allo-HSCT patients^16–18^. Therefore, a mostly homogenous cohort of allo-HSCT patients under severe and life-threatening conditions has been investigated.

Our ICU admission rate of 14.9% was low compared to previous published studies. In addition, the median time from HSCT to ICU admission was with 8.5 (IQR 0.0–16) days also shorter than previously described. The discrepancy is mainly caused by the exclusion of allo-HSCT patients in late course after transplantation. The ICU admission of all allo-HSCT patients after transplantation in our center (33%) compared with other centers has shown similar admission rates^3,19,20^. As observed for other critically ill patients, ICU survival of allo-HSCT recipients has improved over the last decades. The ICU survival rate of 56.4% and in hospital mortality of 58.9% compares favorably with previous reports^8,10,12,21,22^. Unfortunately, 1-year survival with 23.1% remains poor and is in the range previously reported.

Consistent with other reports, several factors were predictive of mortality on ICU during the ICU admission including invasive mechanical ventilation, mean arterial pressure norepinephrine amount and heart rate^4,19,23^. Sex, age, body mass index (BMI) and disease related factors have shown no significant role in determining ICU outcome. Among all clinical and laboratory parameters lactate was the most significant one. Unfortunately, none of the tested prognostic scores (SOFA, SAPS II, APACHE II and APACHE IV) includes lactate as a parameter. Increased blood lactate levels have been related to morbidity and mortality in different patient groups^24,25^. Thus, lactate measurement might be an additional laboratory parameter for predicting ICU mortality and evaluated in further studies. Measurement of procalcitonin (PCT) at date of admission had no prognostic power in our cohort. Higher levels of PCT during conditioning chemotherapy and around transplantation date are probably triggered by anti-thymocyte gobulin (ATG) as reported by Brodska *et al*. and have no predictive value of future infectious complications^26^. Surprisingly, regarding 1-year overall survival urine output during the first 24 hours after ICU admission was the most significant clinical and laboratory parameter. Acute kidney injury is a risk factor for mortality and has been shown in a number of previously published studies^27–29^. Reduced urine output seems to be highly relevant for short- and long-term survival in allo-HSCT patients on ICU during transplant hospitalization. Our study shows the high relevance of the urine output as a marker of long-term outcome of allo-HSCT patients admitted to ICU during peri-transplant period.

In our cohort the APACHE II score showed best discriminative power predicting ICU mortality followed by APACHE IV score. Unfortunately, the newly developed PICAT score was not valuable predicting mortality on ICU as well as short-and long-term survival of allo-HSCT patients admitted to ICU during peri-transplant period. The PICAT score was developed by inclusion of all ICU patients after allo-HSCT. Bayraktar *et al*. have described an increased mortality with increased level of PICAT score up to 91% with a PICAT score higher than 4^14^, whereby in our cohort patients with a score of more than 4 had a mortality of 46%. Allo-HSCT patients in early and late time after transplantation might face different levels of laboratory parameters, time to ICU from hospital admission as well as influence of relapse, acute and chronic GvHD.

Our study has certain limitations, primarily its retrospective nature. All transplantation- and disease-related factors, clinical and laboratory parameters were collected retrospectively. Second, this is a single-center study and criteria for ICU admission might vary from one to another center partially in a substantial manner. Therefore, uniform criteria for ICU admission, laboratory parameters and prognostic scores should be tested and validated in prospective multicenter studies.

In conclusion, this study demonstrated that a considerable number of patients after allo-HSCT has to be transferred to the ICU during transplantation. Despite ICU survival of allo-HSCT patients improved over the last decades long term outcome still remains poor after ICU discharge. In our cohort of peri-transplant patients the APACHE II score has shown to be superior to all other tested prognostic scores, even to the newly developed PICAT score. Our findings might be helpful for designing prospective multicenter studies and prognostic scores by inclusion of new and established clinical and laboratory parameters. For the development of a prognostic score for critically ill allo-HSCT patients during transplant hospitalization, the inclusion of urine output and lactate should be highly considered. High-risk patients could be identified and treatment performed according to their risk stratification. Additionally, enhanced assessment of mortality of critically ill patients might be helpful in conversations about prognosis with relatives. Furthermore, therapies preventing and treating acute kidney injury as well as research in critical care management may substantially improve patients’ prognosis after allo-HSCT.

## Material and Methods

### Patients

We retrospectively reviewed all consecutive adult patients (>18 years) with hematological malignancy who underwent a first allo-HSCT at our institution and had to be transferred to ICU during peri-transplant period (d-7 to d + 30) between January 2010 and January 2017. For patients with multiple ICU admissions, only the first ICU admission was analyzed. Allogeneic HSCT was performed according to EBMT and JACIE guidelines (https://www.ebmt.org/accreditation/jacie-standards). The study was conducted in accordance with Good Clinical Practice Guidelines and the amended Declaration of Helsinki (1964). The study has been approved by the Landesaerztekammer Rhineland-Palatine Ethics Committee (Approval ID:2018–13837) and the Institutional Review Board waived the need for informed consent.The patients transferred to ICU presented a severe and life-threatening condition necessitating single or combinatory need for mechanical ventilation (MV), renal replacement therapy (RRT) with hemodynamic support, uncontrolled bleedings, strong impairment of vigilance or/and continuous invasive monitoring of vital parameters due to septic shock. Treatment of patients requiring only low dose amount of vasopressors was performed in the transplant unit (norepinephrine <0, 3 mg/h).

Clinical variables like age, sex, size, weight, underlying disease, HSCT parameters, and conditioning regimen, reason for ICU admission have been taken from patient chart. Definitions for myeloablative conditioning are according to the CIBMTR working committee^30^. The standardized calculation of the Hematopoietic Cell Transplant-Co Morbidity Index (HCT-CI) for patients has been introduced in 2015 in our department^31^. For patients before 2015 the HCT-CI score had to be calculated retrospectively from patient chart.

Clinical variables (Glasgow coma scale, non-invasive/invasive mechanical ventilation, renal replacement therapy, fluid balance, urine output, body temperature, heart rate, blood pressure, respiratory rate, peripheral oxygen saturation, norepinephrine consumption) and laboratory values (white blood cells, thrombocytes, hematocrit, international normalized ratio (INR), activated partial thromboplastin time (aPTT), fibrinogen, sodium, potassium, bicarbonate, creatinine, blood urea nitrogen, alanine aminotransferase (ALT), aspartate aminotransferase (AST), bilirubin, lactate dehydrogenase (LDH), albumin, total protein, blood sugar, C-reactive protein, procalcitonin, lactate, pH, pO_2_, pCO_2_) during the first 24 hours on ICU were monitored. Based on the recorded variables the SOFA, the SAPS II, the APACHE II and IV and the PICAT score have been calculated at admission to ICU as previously described^6,7,14,32,33^.

Overall survival is defined as the time from the day of hematopoietic stem cell transplantation to death from any cause. The minimal and median follow-up time for ICU survivors was 14 and 38 months, respectively.

### Statistical analysis

Categorical variables were reported as numbers and percentages, and continuous variables as median and interquartile range. Correlations with ICU mortality were evaluated using the Chi-square test for categorical data and the Mann-Whitney U test for continuous variables. For the 1-year survival a binary logistic regression model was used, this was possible as there was no censoring before one year. Survival curves were obtained using the Kaplan-Meier method. They were compared using the log-rank test and a Bonferroni- Holm post-hoc analysis. *P* < 0.05 was deemed to indicate statistical significance. To assess each prognostic score, discrimination was analyzed by obtaining the area under the receiver operating characteristic curve (AUC). Comparisons between two curves were tested for statistical significance by using the method described by Hanley and Mc Neil^34^. The statistical analyses were performed using the IBM® SPSS® Statistics version 23 and GraphPad Prism® version 5.
